# Sexually dimorphic production of interleukin‐6 in respiratory disease

**DOI:** 10.14814/phy2.14459

**Published:** 2020-05-30

**Authors:** Karosham D. Reddy, Sandra Rutting, Katrina Tonga, Dikaia Xenaki, Jodie L. Simpson, Vanessa M. McDonald, Marshall Plit, Monique Malouf, Razia Zakarya, Brian G. Oliver

**Affiliations:** ^1^ School of Life Sciences University of Technology Sydney Sydney NSW Australia; ^2^ Respiratory Cellular and Molecular Biology Woolcock Institute of Medical Research University of Sydney Sydney NSW Australia; ^3^ St Vincent’s Hospital Sydney and St Vincent’s Clinical School University of New South Wales Darlinghurst NSW Australia; ^4^ Priority Research Centre for Healthy Lungs Faculty of Health and Medicine University of Newcastle Callaghan NSW Australia

**Keywords:** fibroblasts, inflammation, respiratory disease, sex‐specific response

## Abstract

Diverging susceptibility and severity in respiratory diseases is prevalent between males and females. Sex hormones have inconclusively been attributed as the cause of these differences, however, strong evidence exists promoting genetic factors leading to sexual dimorphism. As such, we investigate differential proinflammatory cytokine (interleukin (IL)‐6 and CXCL8) release from TNF‐α stimulated primary human lung fibroblasts in vitro. We present, for the first time, in vitro evidence supporting clinical findings of differential production of IL‐6 between males and females across various respiratory diseases. IL‐6 was found to be produced approximately two times more from fibroblasts derived from females compared to males. As such we demonstrate sexual dimorphism in cytokine production of IL‐6 outside the context of biological factors in the human body. As such, our data highlight that differences exist between males and females in the absence of sex hormones. We, for the first time, demonstrate inherent in vitro differences exist between males and females in pulmonary fibroblasts.

## Graphical abstract

Current understanding of the sex differences in respiratory disease is incomplete. We identify for the first time, a difference in immunological response between men and women with respiratory disease diagnosis. Hence, we show a differential response exists outside the context of hormones.

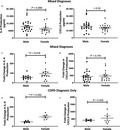

## 1 INTRODUCTION

Sexual dimorphism occurs in various pathologies such as cardiovascular disease, cancer, and respiratory conditions (Alexander, Dasinger, & Intapad, 2011; Lopes‐Ramos et al., 2018; Raghavan & Jain, 2016); influencing pathogenesis, progression, and response to treatment. This phenomenon is prominent in respiratory illnesses such as chronic obstructive pulmonary disease (COPD) and asthma. There is evidence that females have a preponderance for developing COPD, but the mechanism is not understood (Eisner et al., 2005; Tam et al., 2016). Furthermore, prepubescent males have higher prevalence of asthma (Almqvist, Worm, Leynaert, & ‘GENDER’, W. G. O. G. L. W., 2008). This susceptibility is attributed to male airway growth lagging that of the parenchyma (dysanapsis) restricting expiratory rate, whereas females have accelerated lung growth and increased airway size (Almqvist et al., 2008). Therefore, clear differences exist between the sexes in the prevalence of respiratory disease, however, the exact mechanism remains unknown.

One of the best‐described examples of sex difference in a disease is in asthma where a shift occurs during puberty; female asthma incidence increases over‐and‐above males (Almqvist et al., 2008). Age‐associated changes in sex‐hormone levels are often attributed as the cause. In fact, estrogen and testosterone have opposing effects on the immune system; immunocompetence and suppression, respectively (Fish, 2008). Similarly, sex hormones have been ascribed to cause differences between male and female COPD phenotypes; affecting oxidative stress pathways and airway remodeling (Tam et al., 2016).

However, sex hormones cannot fully explain sex differences in respiratory disease. Sexual dimorphism occurs before gonadal development; well before sex‐hormone production (Deegan & Engel, 2019; Werner et al., 2017). Males and females demonstrate distinct responses to their environment at this early stage, reflecting clear sexual identity informed by genetic factors (Deegan & Engel, 2019). Recent GWAS evidence suggests X‐chromosome miRNA contributes to asthma onset at different stages of development (Ferreira et al., 2019). Therefore, a hormone‐independent mechanism can drive sex differences. It remains unclear whether nonhormonal factors significantly contribute to disease later in life.

Tumor necrosis factor‐alpha (TNF‐α) is recognized as a potent proinflammatory cytokine in various respiratory diseases. TNF‐α is well‐known to stimulate production of interleukin (IL)‐6 and C‐X‐C motif ligand 8 (CXCL8), prominent chemo‐attractant cytokines which induce neutrophil infiltration and activation, driving inflammation in the lungs (Lundblad et al., 2005). This study aimed to investigate if IL‐6 and CXCL8 production differ in pulmonary fibroblasts derived from male and female patients in‐vitro when stimulated by TNF‐α.

## 2 MATERIALS AND METHODS

### 2.1 Patients

Primary fibroblasts were isolated from 36 samples of lung parenchyma from patients with a variety of diagnoses. Each diagnosis was made by thoracic physicians according to current guidelines. Protocols were submitted to and approved by a human research ethics committee and prior written and informed consent was obtained from patients under approval by code #X14‐0045. Patient demographics are summarized in Table 1.

**TABLE 1:** Summary patient demographics

	Male	Female
*n*	21	15
Mean age (±*SD*)	58.3 (±12.1)	53.2 (±15.3)
Mean FEV_1_/FVC (±*SD*)	0.61 (±0.28)	0.48 (±0.21)
Smokers/nonsmokers/unknown	16/3/2	8/3/4
Pathology		
COPD (GOLD stage 4)	*n* = 6	*n* = 6
Idiopathic pulmonary fibrosis	*n* = 8	–
Thoracic malignancy	*n* = 4	*n* = 3
Bronchiolitis	*n* = 1	*n* = 3
Pulmonary hypertension	*n* = 1	–
Bronchiectasis	–	*n* = 1
Eisenmenger syndrome	–	*n* = 1
Pneumonitis	–	*n* = 1
No diagnosis	*n* = 1	–

### 2.2 Cell culture

Primary lung fibroblasts were isolated from human lung tissue, as previously described by (Krimmer, Ichimaru, Burgess, Black, & Oliver, 2013). Cells were grown in vitro, seeded at a density of 6.2 × 10^−4^ cells/ml in 12‐well plates in Dulbecco's Modified Eagles Medium (DMEM) (Gibco) containing 5% fetal bovine serum (FBS), 25 mM Hepes buffer (Gibco), and 1% antibiotic‐antimycotic (Gibco) at 37°C/5% CO_2_. Once cells reached 80% confluency, they were serum starved in DMEM supplemented with 0.1% bovine serum albumin (BSA) (Sigma‐Aldrich), 25 mM Hepes buffer, and 1% antibiotic‐antimycotic for 24 hr prior to stimulation. Fibroblasts cultures between passages 2 and 4. The use of early passages attempts to ensure that cell health and processes are maintained as much as possible. Mycoplasma testing was completed on all cell‐lines and returned a negative result.

### 2.3 Cell Stimulation with TNF‐α

Isolated primary fibroblasts were stimulated with TNF‐α (1 ng/ml) (ThermoFisher #T0157) or vehicle control (0.1% BSA) for 24 hr. All cells were incubated at 37°C/5% CO_2_ for 48 hr. Cell‐free supernatants were collected and stored at −20°C until further analysis. IL‐6 and IL‐8 production were measured in cell‐free supernatant by ELISA.

### 2.4 Measurement of IL‐6 and CXCL8 levels

Sandwich ELISA was used on cell‐free supernatants to measure the level of IL‐6 and CXCL8 cytokines as described by (Rutting et al., 2018).

### 2.5 Statistical analysis

Statistical analysis was completed using GraphPad Prism version 8 software (GraphPad Software). Comparisons were carried out on the data by Student's parametric two‐tailed *t*‐test. All data on figures are presented as mean ± standard error of the mean (*SEM*). Statistical significance was determined at *p* < .05.

## 3 RESULTS

### 3.1 Fibroblasts from male and female donors demonstrate different responses To TNF‐Α stimulation

**FIGURE 1:**
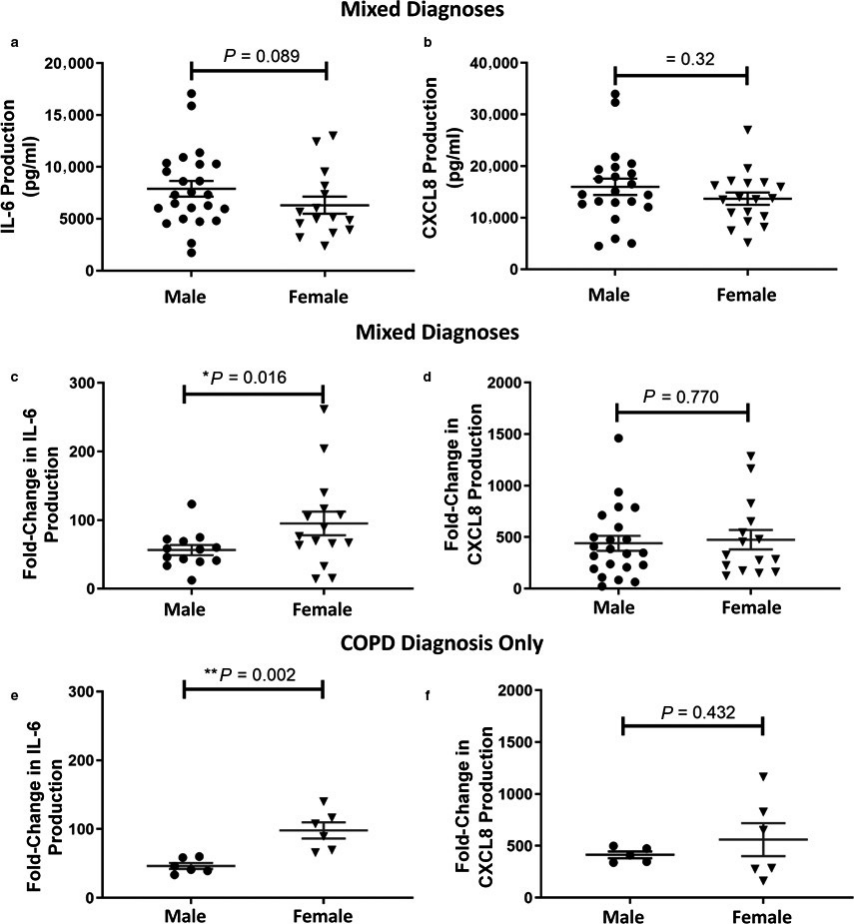
Effect of TNF‐α on IL‐6 and CXCL8 production from pulmonary fibroblasts. Cells were treated with TNF‐α (1 ng/ml) for 24 hr. TNF‐α induced IL‐6 and CXCL8 production (a and b). Fold‐change determined by comparison of TNF‐α stimulated to untreated cells (c and d). Fold‐change determined by comparison of TNF‐α stimulated to untreated cells from patients with COPD (e and f). Data are represented as mean ± *SEM* and analyzed using a Student's parametric two‐tailed *t*‐test; *n* = 5–21. Statistical significance is indicated by *p < .05 and **p < .01

IL‐6 and CXCL8 production was measured in cell‐free supernatant by ELISA. No difference in baseline production of either cytokine was seen by pulmonary fibroblasts between male and female patients; IL‐6:133.0 ± 17.06 pg/ml versus 98.80 ± 21.78 pg/ml and CXCL8: 36.57 ± 4.22 pg/ml versus 33.74 ± 5.32 pg/ml, respectively (Figure 1a and b). Similarly, no significant difference was observed between male and female derived fibroblast when stimulated by TNF‐α. However, female derived fibroblasts produced a greater fold‐change from baseline increase in IL‐6 production than males; 95.15 ± 17.27 versus 53.94 ± 29.94 (*p* = .016), respectively (Figure 1c). This effect was observable irrespective of disease. Conversely, no difference was observed between the sexes when CXCL8 was investigated in the same manner (Figure 1d). These results were reflected in the subpopulation of COPD only diagnoses, where fibroblasts derived from female patients showed greater fold‐change in IL‐6 production compared to male derived cells; 98.11 ± 11.70 versus 46.49 ± 4.38 (*p* = .002), respectively (Figure 1e). Regardless of respiratory diagnosis, females produce almost double the IL‐6 production due to TNF‐α stimulation. Upon removal of the fibroblasts from males with an IPF diagnosis, the trend toward greater fold‐IL‐6 production from females was maintained, although not significant; *p* = .061 (data not shown). No differences in TNF‐α induced CXCL8 production were observed suggesting cytokine‐specific sexual dimorphism can occur.

## 4 DISCUSSION

We show a sexually dimorphic expression pattern exists in fibroblasts removed from the human body. These cells were removed from natural biological influences of hormones and grown for 2–3 months in vitro, indicating an intrinsic mechanism contributes to the sexually dimorphic production of IL‐6 in respiratory disease. Female cells were more liable toward a greater induction of cytokine production compared to males, which may be associated with their generally worse prognosis in respiratory disease. Therefore, our data suggest a hormone‐independent regulatory mechanism exists between the sexes. This is the first study to demonstrate this phenomenon in vitro in primary human pulmonary fibroblasts.

A similar phenomenon has been observed in both murine and human in‐vivo studies. When exposed to ozone, female mice demonstrated increased *IL‐6* expression, among other inflammatory genes, compared to males (Cabello et al., 2015). Although, this difference occurs outside of a disease state. Furthermore, IL‐6 among other cytokines are reported to differ between the sexes in COPD (de Torres et al., 2011). This, when compared with our results supports the potential of disease affecting male and female immune responses differently. These studies highlight sexually dimorphic gene regulation in response to stimuli which may contribute to respiratory disease processes. Hence, these studies in conjunction with our findings suggest sex differences may be driven by an internal cellular mechanism.

Our data support studies which indicate tissue‐specific sexually dimorphic regulation. This is well‐described for the gonads; the primary tissue where sex differentiation exists. This bias is evolutionarily conserved through multiple species and taxa from flies to primates, recognized at the mRNA level (Ober, Loisel, & Gilad, 2008). Importantly, sexually dimorphic expression is reported for autosomal genes, indicating a complex regulatory network is contributing to sex differences. Importantly, the sexually dimorphic expression has also been reported for genes encoded on autosomes in a tissue‐specific manner. The evolution of these genes is asserted to be driven by sex‐specific pressures, increasing sex‐bias over time (Reinius et al., 2008). Therefore, the same exposure would result in different gene sets being utilized between the sexes.

It is important to acknowledge that these differences may be a consequence of the influence of epigenetic marks induced by sex hormones which have been maintained in vitro (Nugent et al., 2015). Fibroblasts derived from various locations in the body have been characterized to express sex steroid hormones receptors, including estrogen receptor alpha (ERα) (Mukudai et al., 2015). Further, a dynamic interplay between hormones and epigenetic patterns has been established. In fact, estrogen is described to exert an epigenetic influence on gene expression (Asai et al., 2001; Zhang & Ho, 2011). TNF‐α signaling is influenced by estrogen, subsequently impacting the immune response (Song, Kim, Kim, Lee, & Surh, 2019). Thus, epigenetic patterns imposed by sex hormones during the patient's lives could be maintained ex vivo, and influence the observed sexual dimorphic pattern reported in this study. However, the locality and longevity of such epigenetic marks is yet to be understood. Most studies looking at this interplay focus on the brain with limited work in the lung. It is prudent for future investigation to focus on the hormone‐epigenetic interplay, as this will offer insight into the complex genetic‐epigenetic mechanisms in disease.

Our study has limitations. Smoking history was only available for 83% of patients, the majority were ex‐smokers (*n* = 24), and few were never‐smokers (*n* = 6). However, the population diagnoses include smoking and nonsmoking‐related diseases, reducing the likelihood of smoking functioning as a determining factor. Nonetheless, the potential contribution of smoking requires further investigation. Females generate an increased immune response to tobacco (Kynyk, Mastronarde, & McCallister, 2011). However, the driving mechanism remains unknown. Tobacco alters the epigenome in a sex‐specific manner, presenting a broader mechanism of action (Ladd‐Acosta et al., 2016). The X‐chromosome contains the largest set of immune‐related genes, with those that escape X‐inactivation possibly contributing to this phenomenon. As such, the regulatory genome is sexually dimorphic (Ober et al., 2008), necessitating careful investigation to determine the mechanism for sex differences in gene regulation.

## 5 CONCLUSIONS

Here, we present for the first time sexually dimorphic IL‐6 production in‐vitro. We speculate this difference is driven by either conserved genetic predisposition or epigenetic regulation of transcription. However, it is possible this effect is due to a continued hormone imprint on the genome; therefore, a detailed investigation is required. Our study shows that differential regulatory mechanisms exist between the sexes and is maintained outside of the body. As such, we highlight the importance of reporting sexual dimorphism in all investigations.

## CONFLICT OF INTEREST

The authors have nothing to disclose.

## AUTHORS’ CONTRIBUTIONS

KDR, BGO, and RZ conceived the idea. KDR, SR, KT, DX, MP, and MM contributed to data acquisition. KDR, BGO, JS, and VM performed, verified and discussed data analysis and interpretation. All authors discussed and contributed to the drafted manuscript for intellectual content.
